# Alteration of the intestinal microbiota associated with the development of nonalcoholic steatohepatitis and sarcopenia in SHRSP5/Dmcr

**DOI:** 10.1007/s12223-025-01283-3

**Published:** 2025-06-10

**Authors:** Taketo Fukuoka, Shusei Yamamoto, Koki Honma, Moe Fujii, Hinako Nakayama, Sora Kirihara, Kazuyoshi Gotoh, Shuma Tsuji, Yuki Kawai, Haruka Tago, Yuka Kono, Kunihiro Sonoda, Kazuya Kitamori, Shogo Watanabe

**Affiliations:** 1 Department of Medical Laboratory Science, Graduate School of Health Sciences, Okayama University, 2-5-1, Shikata-cho, Kita-ku, Okayama-shi, Okayama 700-8558 Japan; 2 Department of Medical Laboratory Science, Faculty of Health Sciences, Okayama University, 2-5-1, Shikata-cho, Kita-ku, Okayama-shi, Okayama 700-8558 Japan; 3 Department of Medical Technology, Ehime Prefectural University of Health Sciences, 543, Takoda, Tobe-cho, Iyo-gun, Ehime 791-2101 Japan; 4 College of Human Life and Environment, Kinjo Gakuin University, 2-1723, Omori, Moriyama-ku, Nagoya-shi, Aichi 463-8521 Japan

**Keywords:** Nonalcoholic steatohepatitis, Sarcopenia, Intestinal microbiota, *Bacteroides*, *Ruminococcus*

## Abstract

Sarcopenia, characterized by skeletal muscle atrophy, was previously considered age-related; however, it is also associated with other diseases. Nonalcoholic fatty liver disease (NAFLD) is known to cause sarcopenia, and its complications have been reported to affect prognosis. The intestinal microbiota of patients with NAFLD or sarcopenia has been found to be altered compared to that of healthy individuals. However, the alterations that occur when both diseases coexist in humans or experimental animals remain unclear. Therefore, this study aimed to determine the intestinal microbiota changes associated with NAFLD with sarcopenia in SHRSP5/Dmcr rats at the time of concomitant disease. Fecal samples were collected from the rectum of SHRSP5/Dmcr rats fed a normal diet (non-NAFLD and non-Sarcopenia, *n* = 5) or a high-fat and high-cholesterol diet (NAFLD and Sarcopenia, *n* = 5) for 20 weeks, and subjected to 16S rRNA analysis. In the NAFLD and sarcopenia group, the diversity of the intestinal microbiota was reduced; further, the bacterial species reported in patients with NAFLD or sarcopenia were also changed. At the family level, the abundances of Akkermansiaceae, Bacteroidaceae, and Tannerellaceae were significantly higher whereas Ruminococcaceae and Lactobacillaceae were decreased in the NAFLD and sarcopenia group. At the genus level, the abundances of *Akkermansia*, *Bacteroides*, *Ruminococcus*, and *Parabacteroides* were significantly higher whereas the abundance of *Lactobacillus* was significantly decreased in the NAFLD and sarcopenia group. Overall, these findings help improve the existing understanding regarding the intestinal microbiota changes observed in conditions where NASH and sarcopenia co-occur.

**Supplementary Information:**

The online version contains supplementary material available at 10.1007/s12223-025-01283-3.

## Introduction

Various bacteria coexist in the intestines of mammals, including humans, and this ecosystem is known as the intestinal microbiota. The intestinal microbiota is controlled by the host immune system, inhibits pathogenic bacterial colonization in the intestines, and facilitates the metabolism of carbohydrates and lipids. This symbiotic relationship has beneficial effects on the host (Adak and Khan 2019). However, this symbiosis can be affected by antibiotics, lifestyle changes, aging, environmental factors, and/or genetic predisposition. These changes and disruptions in the intestinal microbiota composition are referred to as dysbiosis (Weiss & Hennet 2017). Dysbiosis is a risk factor for developing various diseases, such as inflammatory bowel disease, type 2 diabetes, and Parkinson’s disease (Clemente et al. 2012; Hsiao et al. 2013; Sharon et al. 2014).

Nonalcoholic fatty liver disease (NAFLD), one of the most common chronic liver diseases worldwide, has also been reported to be associated with dysbiosis (Boursier & Diehl 2015; Mouzaki et al. 2013; Raman et al. 2013; Zhu et al. 2013). NAFLD presents as hepatic steatosis independent of alcohol consumption and is histologically divided into nonalcoholic fatty liver and nonalcoholic steatohepatitis (NASH) (Nd 2019; Rinella 2015). NASH is characterized by hepatic steatosis, inflammatory cell infiltration, and liver fibrosis. Further, patients with NASH have an increased mortality rate from cardiovascular disease (Targher et al. 2016; Wong et al. 2011). However, no effective treatment for NAFLD/NASH has been established, and elucidating its pathological mechanism and identifying suitable therapeutic targets is thus an urgent need. Recently, sarcopenia, which causes skeletal muscle atrophy, has been associated with liver fibrosis in patients with NASH (Koo et al. 2017; Lee et al. 2015).

Sarcopenia is a muscle-wasting disease that causes a simultaneous decrease in muscle mass and strength. Sarcopenia is classified into two categories, as reported by the European Working Group on Sarcopenia in Older People: primary sarcopenia and secondary sarcopenia (Cruz-Jentoft et al. 2010). The former is defined as age-related sarcopenia, whereas the latter is caused by various diseases, such as malignant tumors, diabetes, chronic kidney disease, extreme lack of exercise, and a bedridden state (Bauer et al. 2019). Inflammatory cytokines, such as TNFα and IL-6, are produced by inflammatory cells and adipose tissue, in cases of chronic inflammation caused by lack of exercise, aging, diabetes, and other diseases. These inflammatory cytokines induce sarcopenia by activating NFκB through its receptors in skeletal muscle, thereby increasing the muscle proteolytic system, which includes the ubiquitin ligases MuRF1 and Atrogin1 (Petersen & Pedersen 2005). Decreased physiological activity in sarcopenia is considered a problem because it worsens life prognosis (Senior et al. 2015). Recently, age-related changes in the intestinal microbiota have been suggested to be associated with various muscle-wasting diseases, including sarcopenia (Collins et al. 2016; Kang et al. 2021; Siddharth et al. 2017; Wang et al. 2022). However, the effects of intestinal microbiota changes in the context of NAFLD/NASH on the skeletal muscle remain to be verified. In this study, we used stroke-prone spontaneously hypertensive rats 5 (SHRSP5/Dmcr) to determine changes in and characteristics of the intestinal microbiota under the co-occurrence of NASH and sarcopenia.

## Materials and methods

### Animal models and diets

In this study, we investigated the rat intestinal microbiota using a sample from our previous study (Yamamoto et al. 2023). The conditions and diets of the rats were as follows:

Nine-week-old male SHRSP5/Dmcr rats (*n* = 10) were obtained from the Disease Model Cooperative Research Association (Kyoto, Japan). SHRSP5/Dmcr rats, established from Wistar Kyoto rats, have been reported to develop NASH following a high-fat and high-cholesterol (HFC) diet (Kitamori et al. 2012). The rats were provided water and a stroke-prone (SP) diet (control chow) ad libitum to acclimatize them to the environment during the first week. At 10 weeks of age, the SHRSP5/Dmcr rats were divided into the SP and HFC groups (*n* = 5 rats each); the former were fed an SP diet, whereas the latter were fed an HFC diet for 20 weeks. The SP (20.8% crude protein, 4.8% crude lipid, 3.2% crude fiber, 5.0% crude ash, 8.0% moisture, and 58.2% carbohydrate) and HFC (9.6% crude protein, 24.0% crude lipid, 1.5% crude fiber, 2.3% crude ash, 3.7% moisture, 26.9% carbohydrate, 25.0% palm oil, 5.0% cholesterol, and 2.0% cholic acid) diets were purchased from Funabashi Farm (Chiba, Japan).

All animal experiments were performed in strict accordance with the recommendations of the Standards of Care and Management of Laboratory Animals and Relief of Pain published by the Japanese Ministry of Environment (2006). In this study, all rats were maintained in a temperature- (24 °C ± 2 °C) and humidity-controlled (55% ± 5%) facility with a 12-h light/dark cycle. This study was approved by the Animal Experiment Committee of Okayama University (approval No. OKU-2021208).

### Sample collection and analysis of intestinal microbiome

The rectum of 30-week-old SHRSP5/Dmcr rats that were fasted overnight was harvested. Fecal samples were collected from the excised rectum into collection tubes. The collected feces were frozen and stored at −80 °C as specimens for intestinal microbiota analysis. After collecting fecal samples from all ten individuals, the frozen fecal samples were sent to Biological Engineering Co., Ltd. (Kanagawa, Japan) for 16S rRNA V3/V4 region sequencing. Data processing was performed using the QIIME2 pipeline (v. 2021.11.0). Taxonomy was assigned with the SILVA 132 97% database. Shannon index, Faith’s phylogenetic diversity, and principal component analysis (PCoA) of UniFrac-weighted distances were calculated using QIIME2 plug-ins. PREMANOVA between different groups was used to statistically test QIIME2 plug-ins. The Mann-Whitney *U* test was used to compare the relative abundances and alpha diversity indices. Relative abundance of each genus was calculated by dividing the number of reads assigned to that genus by the total number of quality-filtered reads in the same sample, and is reported as a proportion of the sample’s microbial community. Sequencing depth for the HFC group ranged from 20,628 to 27,118 reads (mean 23,867 ± 2703), and for the SP group from 11,260 to 20,228 reads (mean 16,838 ± 3127). Importantly, species accumulation curves for all samples plateaued by ~5000 reads, suggesting that sequencing depth was likely sufficient for reliable diversity analysis (Supplementary Figure). Raw, demultiplexed reads were imported into QIIME 2 and denoised using the |q2‐dada2| plugin (|denoise‐single|), with truncation at 250 bp and chimera removal by the consensus method.

## Results

### α-Diversity

**Fig. 1:**
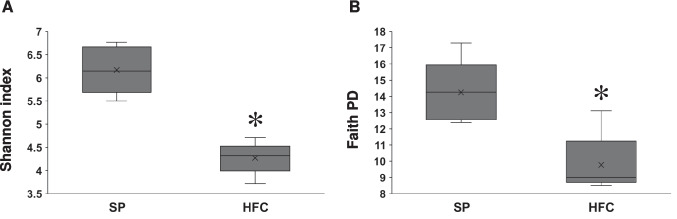
α-Diversity index. The alpha diversity indices (**A**) Shannon index and (**B**) Faith’s phylogenetic diversity were decreased in the HFC group compared to those in the SP group. Mann-Whitney *U* test, *p < 0.05

Compared with the SP group, the Shannon index and Faith’s phylogenetic diversity were significantly lower in the HFC group (Figure 1, Mann-Whitney *U* test, *p* < 0.05).

### Intestinal microbiota

**Fig. 2:**
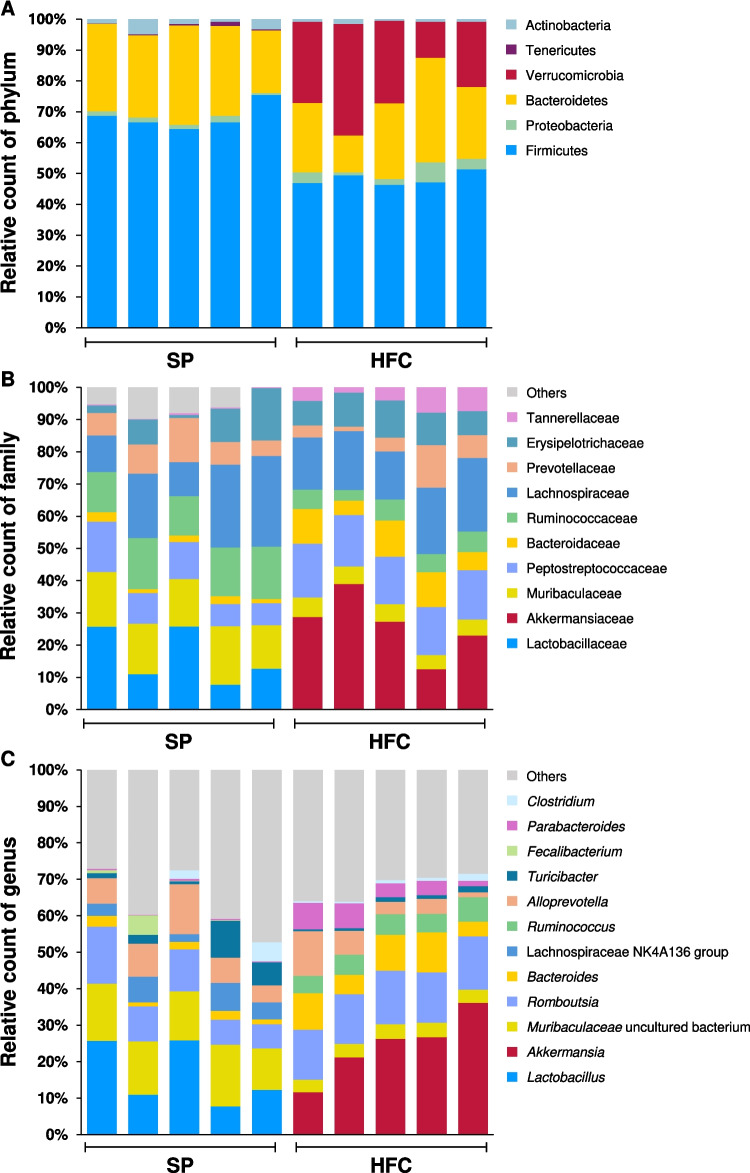
Distribution of gut flora in each individual. The distribution of intestinal microbiota from phylum to genus level was altered in the HFC group compared to the SP group. Stacked columns in each group show the average abundance of a particular family and genus as a percentage of the total. **A** Phylum-level distribution of intestinal microbiota. **B** Family-level distribution of intestinal microbiota. Bacteria with an average abundance of less than 7% are included in others. **C** Genus-level distribution of intestinal microbiota. Bacterial genera with an average abundance of less than 5% are included in others

In this study, 9 bacterial phyla, 65 families, and 127 genera were assigned as bacteria forming the intestinal microbiota in the SP and HFC groups. Comparing the phylum levels of intestinal microbiota in the SP and HFC groups, the relative abundances of Proteobacteria and Verrucomicrobia significantly increased in the HFC group. In contrast, the relative abundance of Firmicutes, Tenericutes, and Actinobacteria was significantly decreased in the HFC group (Figure 2A).

**Fig. 3:**
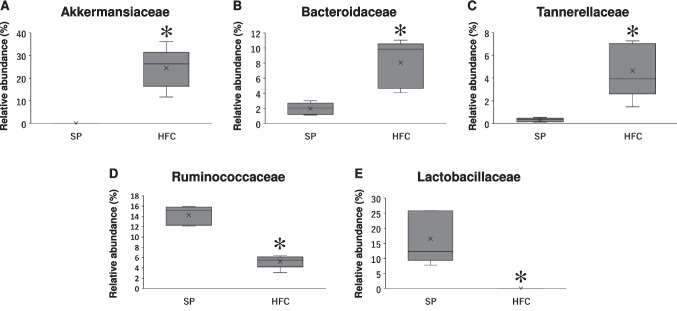
Group comparison of intestinal microbiota: family level. The relative abundance of each family was calculated by dividing the number of reads assigned to that genus by the total number of quality-filtered reads in the same sample. This value is reported as a proportion of the sample’s microbial community

At the family level, the abundances of Akkermansiaceae, Bacteroidaceae, and Tannerellaceae were significantly higher in the HFC group than in the SP group (Figures 2B, 3A–E). In contrast, the abundances of Ruminococcaceae and Lactobacillaceae were decreased significantly in the HFC group (Figures 2B and 3D and E).

**Fig. 4:**
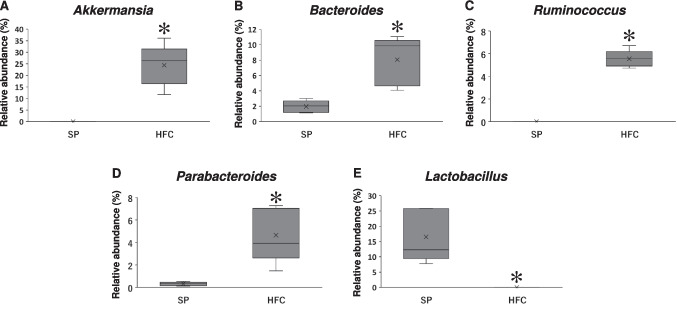
Group comparison of intestinal microbiota: genus level. The relative abundance of each family was calculated by dividing the number of reads assigned to that genus by the total number of quality-filtered reads in the same sample. This value is reported as a proportion of the sample’s microbial community

At the genus level, the abundances of *Akkermansia*, *Bacteroides*, *Ruminococcus*, and *Parabacteroides* were significantly higher in the HFC group than in the SP group (Figures 2C, 4A–D). Further, the abundance of *Lactobacillus* was significantly decreased in the HFC group (Figures 2C, 4E). In contrast, no significant differences were observed in the abundances of *Romboutsia*, *Alloprevotella*, *Muribaculum*, *Turicibacter*, *Clostridium*, *Faecalibaculum*, and *Lachnospira* (Figure 2C).

### β-Diversity

**Fig. 5:**
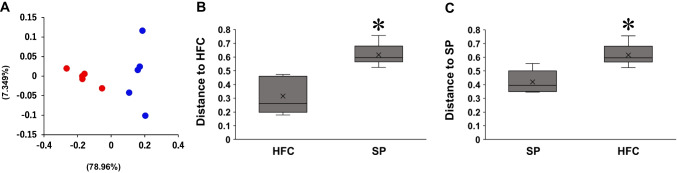
β-Diversity index of SHRSP5/Dmcr rats. The beta diversity index showed a significant difference in the intestinal bacterial composition between the SP and HFC groups. **A** Two-axis principal component analysis (PCoA) of UniFrac-weighted distances. **B** Significance plot generated from UniFrac-unweighted distances. The taxonomic distance of the intestinal bacterial composition in the HFC group is also presented. **C** Significance plot generated from UniFrac-unweighted distances. The taxonomic distance of the intestinal bacterial composition in the SP group is shown. PERMANOVA test, *p < 0.05

In the SP and HFC groups, the UniFrac-weighted distance PCoA showed bias (Figure 5A). The UniFrac-unweighted distance, which indicates the taxonomic distance of the intestinal bacterial composition, showed a significant difference between the two groups (Figure 5B and C).

## Discussion

Alterations in the intestinal microbiota result in changes in the abundance of bacterial taxa; however, these changes can sometimes directly cause disturbances in bacterial balance and decrease diversity or cause dysbiosis (Walker et al. 2011; Weiss & Hennet 2017). Recently, dysbiosis has been reported to influence the development of various diseases. For example, Perlin et al. reported that reduced intestinal microbiota diversity is positively correlated with worsening liver function in patients with severe liver disease (Perlin et al. 2023). Further, the diversity of the intestinal microbiota is positively correlated with skeletal muscle mass, and a decrease in diversity has been observed in patients with sarcopenia due to aging (Park et al. 2022). NAFLD/NASH, a chronic liver disease, is affecting an increasing number of patients worldwide, and the lack of effective treatment options is a major problem. In recent years, sarcopenia has been found to coexist with NAFLD/NASH and has been associated with poor prognosis (Koo et al. 2017; Lee et al. 2015). The co-occurrence of sarcopenia with NAFLD/NASH is considered problematic because of its prognostic relevance; however, effective prevention and treatment methods remain to be established. Despite scattered reports on the intestinal microbiota of patients with NAFLD/NASH, the intestinal microbiota in the presence of concomitant sarcopenia remains unknown. In this study, we focused on the intestinal microbiota alterations in a model of NASH and sarcopenia. The SHRSP5/Dmcr rats used in this experiment developed NASH with sarcopenia when fed an HFC diet for 20 weeks (Yamamoto et al. 2023). In this study, we showed that diversity was significantly reduced in the HFC group (Figures 1 and 5). We have previously shown that the HFC diet group had worse liver function, consistent with reports on patients with severe liver disease (Perlin et al. 2023; Yamamoto et al. 2023).

Although NAFLD includes a wide range of histological changes, ranging from simple hepatic steatosis to advanced liver fibrosis, the influence of the intestinal microbiota has been reported previously (De Minicis et al. 2014; Le Roy et al. 2013; Loomba et al. 2017). Recent evidence has shown an increased abundance of Proteobacteria and a decreased abundance of Firmicutes in the Enterobacteriaceae phylum in patients with NASH and advanced liver fibrosis (Loomba et al. 2017; Wong et al. 2013). Furthermore, at the genus level, an increased abundance of *Bacteroides* is associated with NASH, while an increased abundance of *Ruminococcus* is associated with liver fibrosis in NASH (Boursier et al. 2016). Similar to this evidence, an increased abundance of Proteobacteria and a decreased abundance of Firmicutes were observed in this study when NASH develops (Figure 2A). Alterations in *Bacteroides* and *Ruminococcus* spp. were also consistent with those reported by Boursier et al. (Figures 3, 4). An increase in the abundance of Proteobacteria is a common change in dysbiosis (Walker et al. 2011). The HFC group was associated with severe liver fibrosis; therefore, the decrease in Firmicutes was consistent with that reported in human patients.

The abundance of *Bacteroides* observed in this study was consistent with its increase in the intestinal microbiota of patients with sarcopenia (Ren et al. 2021; Wang et al. 2022). Generally, when NAFLD/NASH progresses in severity, it leads to liver cirrhosis; notably, skeletal muscle atrophy, including sarcopenia, is frequently observed in patients with liver cirrhosis (Wexler 2007). Bacterial genera like *Escherichia*, *Clostridium*, and three types of *Bacteroides* have been reported to be increased in patients with concomitant cirrhosis and sarcopenia (Ren et al. 2021). *Bacteroides* is a genus of gram-negative non-motile anaerobic bacteria, widely distributed in the intestines of mammals, including humans; it obtains energy mainly from carbohydrates and plays an important role in probiotics and mammalian immune responses (Hsu & Kao 2018). Among intestinal bacteria, *Bacteroides* also has a high ability to metabolize tryptophan (Trp) and produce several Trp metabolites, such as 3-methylindole, indole-3-lactic acid, and indole acetic acid (Ninomiya et al. 2020). In skeletal muscles, amino acids are essential for muscle protein synthesis and metabolism; however, aromatic amino acids, such as Trp and branched-chain amino acids, are required for regulating skeletal muscle mass. Ninomiya et al. revealed that skeletal muscle atrophy occurred at a high rate in mice fed a Trp-deficient diet (Russell et al. 2013). Muscle breakdown may thus be accelerated in these mice to replenish the deficient Trp. Notably, Trp levels are decreased in the intestinal flora of patients with sarcopenia. Increased *Bacteroides* abundance in the HFC group may thus lead to skeletal muscle atrophy by altering the host ability to metabolize Trp.

*Ruminococcus* is a genus of bacteria that breaks down various carbohydrates, including cellulose and pectin, to produce alcohol (Christopherson et al. 2014). Patients with NASH have higher blood ethanol concentrations than those in individuals without non-NASH, which is considered a result of increased ethanol-producing bacteria owing to changes in the intestinal microbiota (Zhu et al. 2013). In this study as well, the increased *Ruminococcus* may have increased alcohol production and damaged the host intestinal mucosa, leading to the spillover of inflammation and increased intestinal permeability (Figure 4C). *Lactobacillus* spp. regulate the intestines and improve immunity; as a component of probiotics, it corrects the intestinal environment and improves various diseases (O'Callaghan & O'Toole 2013). In this study, the abundance of Lactobacillaceae and *Lactobacillus* was significantly decreased in the HFC group (Figures 3E, 4E). A decrease in lactic acid bacteria among the intestinal flora can be considered a situation in which the aforementioned probiotics are impaired. Indeed, supplementation with probiotics containing *Lactobacillus* is suggested to improve hepatic lipid accumulation and simultaneously restore skeletal muscle mass and muscle function (Ni et al. 2019). Even in advanced NAFLD/NASH, recovery of liver fibrosis and improvement of liver function can be observed upon *Lactobacillus* supplementation (Velayudham et al. 2009; Zhao et al. 2023). Overall, *Lactobacillus* may suppress and improve NAFLD/NASH and sarcopenia. The decreased abundance of *Lactobacillus* in the HFC group can be considered to indicate a poor control of NASH and sarcopenia progression. Furthermore, Tannerellaceae and *Parabacteroides* were significantly increased in the HFC group (Figures 3C, 4D). In a study of sarcopenia in mice, the abundance of the species *Parabacteroides distasonis* increased in the sarcopenia group (Liu et al. 2023). This finding suggests that our results may also be due to sarcopenia. Additionally, studies have reported that endotoxin-producing Tannerellaceae are significantly higher in the neuroinflammatory group of ovariectomized mice. Feeding rice bran has been shown to reduce these bacteria and alleviate symptoms (Chao et al. 2024). In other words, Tannerellaceae are implicated in disease pathogenesis. However, these alterations in intestinal bacterial composition have not yet been reported to be associated with NAFLD/NASH and sarcopenia and may only be seen in patients with both diseases occurring together.

The intestinal microbiota is now being considered a potential biomarker and therapeutic target. In fact, disease-specific changes in the intestinal microbiome have been reported. For example, decreased Firmicutes and increased *Bacteroides* have been observed in Alzheimer’s disease, while increased *Ruminococcus* has been observed in Crohn’s disease (Liu et al. 2019; Sartor 2010; Vogt et al. 2017). These changes could become biomarkers for diagnosis in the future. In addition, changes in the intestinal microbiota of rodents due to butyric acid intervention and their effects have been reported, and some cases have been developed into clinical studies (Saban Güler et al. 2025). There are also reports of changes in flavonoids affecting the intestinal microbiota in Parkinson’s disease, as well as reports of live biotherapeutic products improving psychological status by improving the intestinal microbiota (Açar et al. 2023; Ağagündüz et al. 2023). The changes observed in this study have the potential to serve as future risk estimators and biomarkers for concurrent sarcopenia in patients with NAFLD/NASH, as well as potential targets for prevention or treatment.

This study had some limitations. In general, fecal material from experimental animals is collected from the cecum whereas that from humans is not. Therefore, in this study, we used feces retained in the rectum to analyze the intestinal microbiota and to make the results more comparable to those of studies on human subjects. This point should be considered when comparing these results with those of other studies because the conditions are different. However, we did not need to consider killing anaerobic bacteria because the collected feces were not dried, obtained after being expelled from the body. SHRSP5/Dmcr rats provide a so-called lean NASH model that does not develop obesity or insulin resistance (Yamamoto et al. 2023). The number of non-obese/underweight patients with NASH is increasing worldwide, with a particularly high prevalence in Asia (Fan et al. 2017). Although there are currently no studies on the intestinal microbiota of non-obese/underweight patients with NASH, *Akkermansia* is considered to be associated with leanness. *Akkermansia* grows in the intestines of mammals, using mucin as its sole nutritional source. The abundance of *Akkermansia* is significantly reduced in obese individuals as well as in obese patients with NASH compared to that in healthy individuals (Everard et al. 2013; Schneeberger et al. 2015). Akkermansiaceae and *Akkermansia* were significantly increased in the HFC group in this study, potentially explaining why SHRSP5/Dmcr rats do not exhibit obesity; however, the cause of their marked increase in the HFC group compared to that in the SP group remains unclear (Figures 2, 3A, 4A).

The human gut microbiota varies by race; thus, different races are unlikely to have the same bacterial composition, even if they have the same disease (Nishijima et al. 2016). Further, the intestinal flora of patients from different countries most closely resembles the bacterial composition of healthy individuals in each country (Karlsson et al. 2013). Therefore, the changes in intestinal flora associated with concurrent NASH and sarcopenia shown in this study do not represent a universally applicable model. However, it could be a useful model for patients and disease development in countries and regions where the intestinal flora composition is similar to that of normal rats.

Decreased diversity of the intestinal microbiota and an increase or decrease in specific bacterial taxa are suggested to influence the co-occurrence of NAFLD/NASH and sarcopenia. In particular, a parallel increase in the abundance of *Bacteroides* and *Ruminococcus* and a decrease in the abundance of *Lactobacillus* are likely characteristics of the co-occurrence of NAFLD/NASH and sarcopenia. These bacteria have been shown to return to normal levels through dietary and drug interventions in other diseases. They could be used as biomarkers and therapeutic targets in the future. The discrepancies in intestinal bacterial composition observed between this study and some referenced studies may be attributed to differences in animal species, disease phenotypes, dietary environments, and sample sequence analyses. Overall, these findings help improve the existing understanding regarding the intestinal microbiota changes observed in conditions where NASH and sarcopenia co-occur.

## Supplementary Information

Below is the link to the electronic supplementary material.

Supplementary file 1: Rarefaction curves for 16S rRNA sequences. (PPTX 87.6 KB)

## Data Availability

The data that support the findings of this study are available from the corresponding author, S. Y., upon reasonable request.

## Abbreviations

NAFLD: Nonalcoholic fatty liver disease
NASH: Nonalcoholic steatohepatitis
SHRSP5: Stroke-prone spontaneously hypertensive rat 5
HFC: High-fat and high-cholesterol
SP: Stroke-prone
PCoA: Two-axis principal component analysis
Trp: Tryptophan
