# Supplementary figures and images for: Alteration of the intestinal microbiota associated with the development of nonalcoholic steatohepatitis and sarcopenia in SHRSP5/Dmcr
Source: Folia Microbiol (Praha). 2025 Jun 10;71(3):585–94. doi: 10.1007/s12223-025-01283-3 (PMC13346266; doi:10.1007/s12223-025-01283-3)

## Slide 1
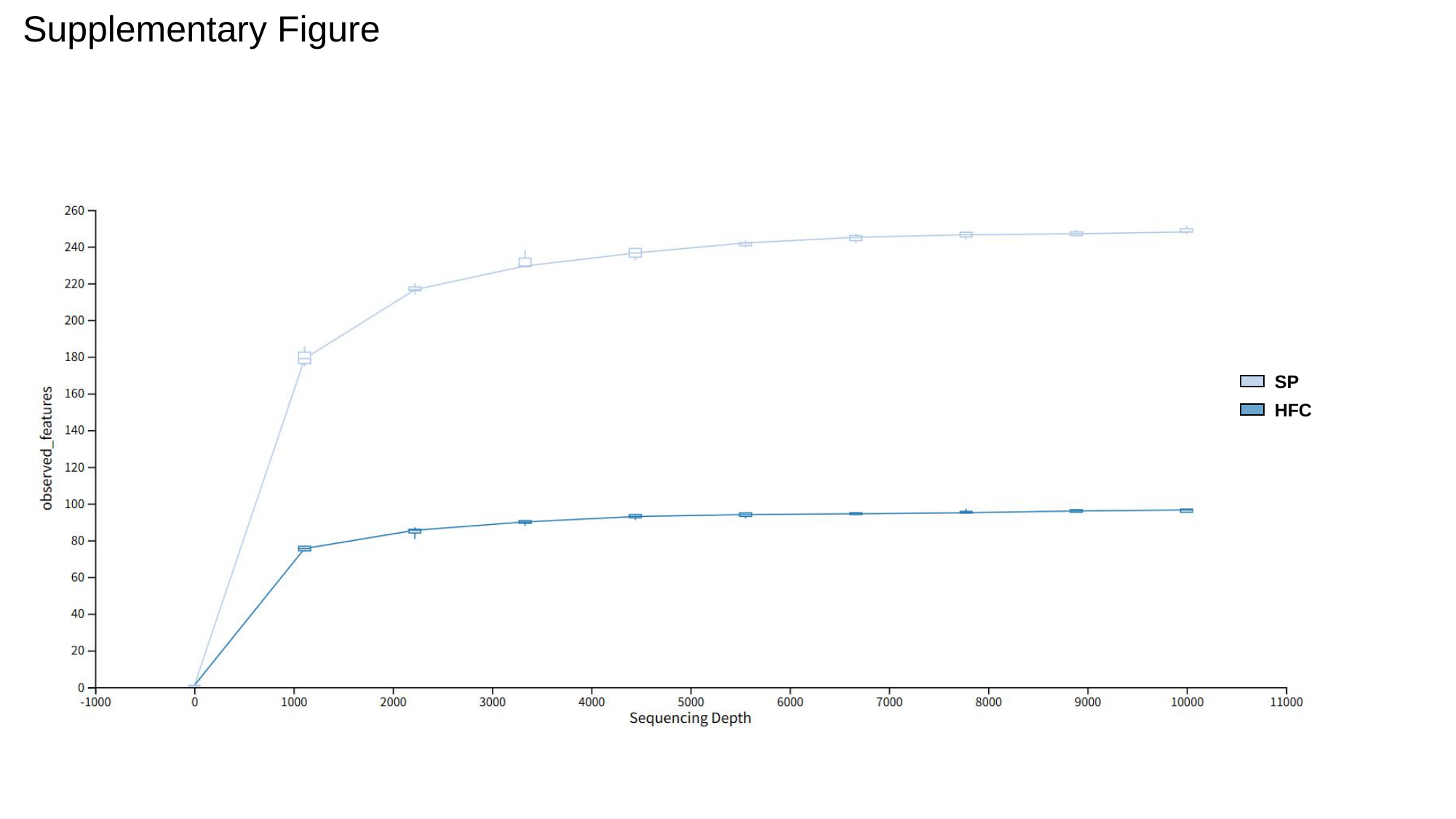

Supplementary Figure
SP
HFC

Supplement: Supplementary file 1 — Rarefaction curves for 16S rRNA sequences. (PPTX 87.6 KB) [file 12223_2025_1283_MOESM1_ESM.pptx]
